# A chromosome scale assembly of the parasitoid wasp *Venturia canescens* provides insight into the process of virus domestication

**DOI:** 10.1093/g3journal/jkad137

**Published:** 2023-06-22

**Authors:** Meng Mao, Tyler J Simmonds, Corinne M Stouthamer, Tara M Kehoe, Scott M Geib, Gaelen R Burke

**Affiliations:** Department of Entomology, University of Georgia, Athens, GA 30602, USA; Tropical Pest Genetics and Molecular Biology Research Unit, USDA-ARS Daniel K Inouye U.S. Pacific Basin Agricultural Research Center, USDA-ARS, Hilo, HI 96720, USA; Oak Ridge Institute for Science and Education, Oak Ridge Associated Universities, Oak Ridge, TN 37830, USA; Department of Entomology, University of Georgia, Athens, GA 30602, USA; Department of Entomology, University of Georgia, Athens, GA 30602, USA; Tropical Pest Genetics and Molecular Biology Research Unit, USDA-ARS Daniel K Inouye U.S. Pacific Basin Agricultural Research Center, USDA-ARS, Hilo, HI 96720, USA; Department of Entomology, University of Georgia, Athens, GA 30602, USA

**Keywords:** *Venturia canescens*, domesticated endogenous virus, parasitoid, nudivirus

## Abstract

The parasitoid wasp *Venturia canescens* is an important biological control agent of stored products moth pests and serves as a model to study the function and evolution of domesticated endogenous viruses (DEVs). The DEVs discovered in *V. canescens* are known as virus-like particles (VcVLPs), which are produced using nudivirus-derived components and incorporate wasp-derived virulence proteins instead of packaged nucleic acids. Previous studies of virus-derived components in the *V. canescens* genome identified 53 nudivirus-like genes organized in six gene clusters and several viral pseudogenes, but how VcVLP genes are organized among wasp chromosomes following their integration in the ancestral wasp genome is largely unknown. Here, we present a chromosomal scale genome of *V. canescens* consisting of 11 chromosomes and 56 unplaced small scaffolds. The genome size is 290.8 Mbp with a N50 scaffold size of 24.99 Mbp. A high-quality gene set including 11,831 protein-coding genes were produced using RNA-Seq data as well as publicly available peptide sequences from related Hymenoptera. A manual annotation of genes of viral origin produced 61 intact and 19 pseudogenized nudivirus-derived genes. The genome assembly revealed that two previously identified clusters were joined into a single cluster and a total of 5 gene clusters comprising of 60 intact nudivirus-derived genes were located in three chromosomes. In contrast, pseudogenes are dispersed among 8 chromosomes with only 4 pseudogenes associated with nudivirus gene clusters. The architecture of genes encoding VcVLP components suggests it originates from a recent virus acquisition and there is a link between the processes of dispersal and pseudogenization. This high-quality genome assembly and annotation represents the first chromosome-scale assembly for parasitoid wasps associated with VLPs, and is publicly available in the National Center for Biotechnology Information Genome and RefSeq databases, providing a valuable resource for future studies of DEVs in parasitoid wasps.

## Graphical Abstract

**Graphical Abstract:**
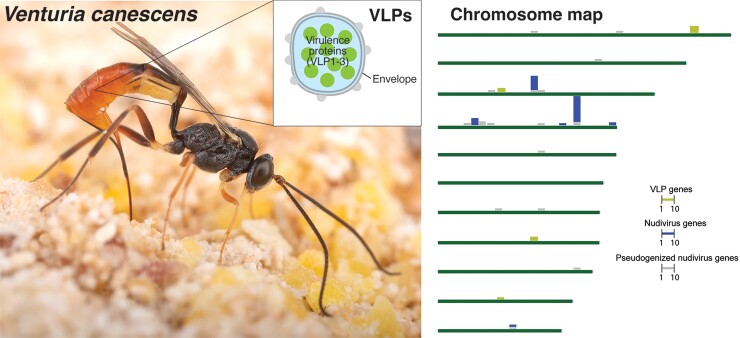

## Introduction

*Venturia canescens* (Hymenoptera: Ichneumonidae) is a synovigenic solitary koinobiont endoparasitoid wasp that plays an important role in integrated pest management programs (Salt 1976; Schöller et al. 1997). It is a parasitoid of stored-product pest insects and can reproduce in sexual and asexual reproductive modes, both of which have made it attractive for studies on physiology, behavior, sex determination, life history traits and trade-offs, and genetic variability (Harvey et al. 2001; Desouhant et al. 2005; Pelosse et al. 2007; Mateo Leach et al. 2012; Amat et al. 2017), and as a model to study the function and evolution of domesticated viruses (Reineke et al. 2006; Pichon et al. 2015; Leobold et al. 2018). *V. canescens* is an effective parasitoid of the larvae of various lepidopteran species including some of the world's most widespread moth pests of stored products, such as *Plodia interpunctella* and *Ephestia kuehniella* (Heinlein et al. 2002; Ozkan et al. 2004).

During parasitism, *V. canescens* lays eggs into moth larvae and uses virus-like particles (VcVLPs) to protect eggs against host defenses (Feddersen et al. 1986; Beck et al. 2000). VcVLPs are produced in the calyx cells during the wasp pupal stage and are released into the calyx lumen, where they attach to egg surfaces during maturation. The VLPs are 100–150 nm electron-dense particles surrounded by viral envelopes (Feddersen et al. 1986). VcVLPs together with bracoviruses (BVs) and Fopius arisanus Endogenous Nudivirus represent three types of nudivirus-derived beneficial domesticated endogenous viruses (DEVs) found in parasitoid wasps (Burke 2019). DEVs represent cases in which a virus genome has become permanently integrated into a wasp genome and viral genes are used to produce viruses or VLPs. Virus-derived genes are inherited by all individual wasps and retained when wasps undergo speciation. VcVLPs are produced using nudivirus-derived components, including envelope proteins, of which genes are hypothesized to be transcribed by an ancestrally viral RNA polymerase (Pichon et al. 2015; Cerqueira de Araujo et al. 2022). Contrary to the well-known BVs with circular double-stranded DNAs packaged into virions, VcVLPs contain virulence proteins of wasp origins and are devoid of packaged nucleic acids (Pichon et al. 2015). The wasp-derived proteins are hypothesized to help cloak the egg against the host's immune system (Feddersen et al. 1986). So far, three wasp derived proteins have been identified as associated with the VcVLPs: *VLP1*, *VLP2*, and *VLP3*. *VLP1* is a putative transmembrane, phospholipid-hydroperoxide glutathione peroxidase-like protein, which may protect against oxidative damage in the wasp (Li et al. 2003). *VLP2* is a RhoGap-like protein, which is hypothesized to interfere with the cytoskeleton of hemocytes (Labrosse et al. 2005; Du et al. 2020). *VLP3* has a neprilysin-like domain. Neprilysin is a metalloendopeptidase that is responsible for the regulation of peptide signaling on the cell surface (Turner et al. 2001), and has been found in many venom-producing wasp species (Colinet et al. 2013; Undheim et al. 2013; Yang et al. 2020).

To date, *V. canescens* is the only wasp in the family Ichneumonidae known to produce nudivirus-derived VLPs, while several ichneumonid wasps are associated with ichnoviruses, indicating VcVLPs might have evolved from an ichnovirus-for-nudivirus replacement event (Pichon et al. 2015). Previous phylogenetic analyses using nudivirus genes from DEVs and pathogenic nudiviruses showed that VcVLPs are derived from *Alphanudivirus*, a distinct genus from the ancestor of BVs (*Betanudivirus*) (Pichon et al. 2015; Leobold et al. 2018; Drezen et al. 2022). Unlike BVs, whose genomic architecture has been investigated comparatively in several wasp species with high-quality genome assemblies (Burke et al. 2014; Gauthier et al. 2021; Mao et al. 2022), the genomic architecture of the VcVLP type of DEVs has only been studied in a single species, *V. canescens*. Fifty-three nudivirus-like genes and several viral pseudogenes have been identified previously in the *V. canescens* genome (Pichon et al. 2015; Leobold et al. 2018; Drezen et al. 2022). However, the current *V. canescens* genome assembly is fragmented and gene annotations were mainly generated for VcVLPs. To gain a better insight into how VcVLPs have evolved after their ancestor was integrated into wasp genomes, we announce a chromosomal scale genome of *V. canescens* with an annotated gene set for both wasp and viral genes. This publicly available genome assembly will continue to facilitate research on the evolution of VLPs, *V. canescens* and comparative genomic studies of wasps.

## Materials and methods

### Wasp samples

Wasps used for genome sequencing and assembly were maintained at the University of Georgia (isolate “UGA”) on its host, *P. interpunctella*. *P. interpunctella* larvae were kept in 32-ounce plastic containers with diet composed of chick starter feed, cornmeal, and glycerol in a 2:2:1 weight ratio (Phillips and Strand 1994). Fourteen-day old *P. interpunctella* larvae were parasitized by *V. canescens* wasps and kept in diet until emergence. Emerged adult wasps were kept in cages and fed with honey and water agar. All cultures were maintained at 26°C, 40–62% humidity, with a 12 h light: 12 h dark photoperiod. Voucher specimens have been deposited in the UGA Collection of Arthropods, University of Georgia, Athens, GA.

### Whole genome sequencing and assembly

High molecular weight DNA was extracted from a single female wasp derived from the colony using the Qiagen MagAttract HMW DNA Kit. Genomic DNA quantity, quality, and purity were assessed using a combination of fluormetric, spectrophoetric, and electrophoretic methods using the Denovix DS-11 and Agilent Genomics FemtoPulse Capillary electrophoresis systems. Once sample size and purity were confirmed to be suitable for library prep, a PacBio SMRTBell library was prepared using the SMRTBell Express Template Prep Kit 2.0. DNA was first sheared to ∼15–20 kb using a Diagenode Megaruptor 2 and then the library prep methods were followed as the manufacturer specifies, with the exception of using a bead-based size selection (3 kb molecule cutoff) using modified SPRI beads in lieu of electrophoresis-based size selection due to low DNA input amount. The prepared library was bound and sequenced at the USDA-ARS Genetics and Animal Breeding Research Unit in Clay Center, Nebraska, USA on a Pacific Biosciences 8M SMRT Cell on a Sequel II system (Pacific Biosciences, Menlo Park, California, USA) beginning with a 2-hour pre-extension followed by a 30-hour movie collection time. After sequencing, consensus sequences from the PacBio Sequel II subreads were obtained by running ccs from pb-bioconda anaconda package. In addition to the HiFi sequencing, a HiC library was prepared from a pool of wasps to ensure sufficient tissue was present to generate high quality proximity ligations. The Arima Genomics HiC kit was used for preparation of the library from formaldehyde-fixed tissue. After HiC proximity ligation, the library was sheared using a Bioruptor Pico and adapted for Illumina sequencing using the Swift Accel 2S Plus library preparation kit. The library was sequenced on a fraction of a lane of Illumina iSeq 100, using 150 base pair paired-end sequencing.

Prior to genome assembly, HiFi reads (SRA accession number: SRR24234441) containing artifact adapter sequences were removed from the HiFi read pool using the program HiFiAdapterFilt (Sim et al. 2022). This filtered read set was assembled into a contig assembly using HiFiASM (Cheng et al. 2021) using the default parameters. The primary contig assembly was scaffolded using the HiC reads (SRA accession number: SRR24234440) generated from the wasp pool. Briefly, HiC reads were mapped to the primary contig assembly using BWA with the -5SP flags to allow for the unique mapping characteristics of the chimeric HiC reads. PCR duplicates were filtered using samblaster, and the subsequent bam file, along with the contig assembly were reformatted to allow visualization and editing in Juicebox using scripts available from Phase Genomics (https://github.com/phasegenomics/juicebox_scripts). The resulting .hic and .assembly files were loaded into Juicebox and manually edited to order and orient contigs into chromosome scale scaffolds. The resulting edited assembly file was used to then output a scaffolded fasta file using juicebox_assembly_converter.py from the same script repository listed above. Blobtools analysis was performed to identify any non-wasp and non-chromosomal contigs and remove them (Laetsch and Blaxter 2017). This was considered the final chromosome set that was then submitted to NCBI for curation and annotation.

### RNA purification and sequencing

RNA was extracted from larval and pupal stages, and ovaries, venom gland, head, thorax, and abdomen from adult females (Table 1). Samples were extracted using the Quick-RNA tissue/insect kit with on-column DNAse treatment (Zymo Research). Samples were then treated with the Ambion TURBO DNA-free kit (Invitrogen). Standard strand-specific Illumina-compatible RNA-Seq libraries were constructed for each sample, and sequenced (2 × 150 bp reads) by Novogene Co. Inc. (CA, USA) using the NovaSeq 6000 system with read yields for each library noted in Table 1.

**Table 1.** RNA-Seq reads from *V. canescens* samples used for annotation.

Sample type	SRA accession	Raw reads sequenced
Adult female head (*N* = 10)	SRR14772634	21.2 million
Adult female thorax (*N* = 10)	SRR14772633	20.8 million
Adult female abdomen (*N* = 10)	SRR14772632	19.0 million
Pupae (mixed stages, *N* = 5)	SRR14772630	20.2 million
Larvae (mixed stages, *N* = 5)	SRR14772628	23.2 million
Ovaries from adult females (*N* = 50)	SRR14772631	20.8 million
Venom glands from adult females (*N* = 50)	SRR14772629	21.2 million

### Genome annotation

Genome annotation was performed using the NCBI Eukaryotic Genome Annotation Pipeline (https://www.ncbi.nlm.nih.gov/genome/annotation_euk/process/). This automated pipeline utilized the RNA-Seq data from this study and existing data in GenBank for *V. canescens* (Pichon et al. 2015), in addition to protein sequences for *V. canescens*, NCBI RefSeq protein sets for *Bombus impatiens*, *Diachasma alloeum*, *Chelonus insularis*, *Harpegnathos saltator*, *Tribolium castaneum*, and *Apis mellifera*, 39,085 other Insecta RefSeq proteins, and 112,623 protein sequences from GenBank derived from the Insecta for gene prediction. Statistics and the evidence used for annotation are available at https://www.ncbi.nlm.nih.gov/genome/annotation_euk/Venturia_canescens/100/. The completeness of the annotated gene set was analyzed by BUSCO v.4.0.5 with the insecta_odb10 lineage dataset (Simão et al. 2015).

### Nudivirus gene annotation

Open Reading Frames (ORFs) from the *V. canescens* genome assembly and annotations generated by NCBI were first searched against the published nudivirus proteins of *V. canescens* as well as a database of nudivirus-like proteins from *C. insularis*, *Microplitis demolitor*, and *Cotesia congregata* using BLASTP (*e* = 0.01) (Burke et al. 2014; Pichon et al. 2015; Gauthier et al. 2021; Mao et al. 2022). ORFs were then searched against the NCBI viral protein database (downloaded November 2021). In addition, previously published pseudogenized nudivirus genes of *V. canescens* were annotated by aligning pseudogene sequences to the genome assembly using MAFFT (Katoh et al. 2002; Leobold et al. 2018; Drezen et al. 2022). All of the identified ORFs or gene annotations were then manually converted into annotated gene models with the *V*. *canescens* jBrowse/Apollo instance on the i5k workspace (https://i5k.nal.usda.gov/available-genome-browsers). A bigWig formatted coverage blot generated from the transcriptome data of ovaries was used to define nudivirus-derived and hypothetical gene transcription boundaries.

### VLP protein identification

To identify each VLP protein sequence in the genome, the amino acid sequences were extracted from the papers in which they were identified (Hellers et al. 1996 for *VLP1*; Reineke et al. 2002 for *VLP2*; Asgari et al. 2002 for *VLP3*). These sequences were searched against the assembled genome using TBLASTN. To look for paralogous genes, BLASTN was used with each *VLP* predicted mRNA sequence and those with the highest nucleotide identity were annotated as described for nudivirus-derived genes.

### Expression analysis of nudivirus genes and VLP genes in ovaries

Raw reads from ovaries of adult females (Table 1) were adapter-trimmed and quality filtered with Trimmomatic v0.36 (program settings: ILLUMINACLIP:2:20:10:1 LEADING:20 TRAILING:20 SLIDINGWINDOW:4:20 MINLEN:36) (Bolger et al. 2014). Expressed genes in ovaries of female adults were determined by read mapping to the assembled genome with HISAT2 (Kim et al. 2015). Transcript assembly and abundance estimation were performed using StringTie and expression value (FPKM) per gene was obtained using Ballgown (Pertea et al. 2016).

## Results and discussion

The *V. canescens* genome assembly using PacBio HiFi reads yielded 11 chromosomes and 56 unplaced scaffolds with an N50 scaffold length of 24.99 Mbp (Supplementary Fig. 1). The overall length of the assembly is 290.8 Mbp (genome assembly coverage = 40×) with only 0.001% of the assembly comprised of sequencing gaps. The G + C content of the genome is 39.6%, which is similar to other parasitoid genomes (Table 2). The BlobPlot also revealed that all the chromosomes and scaffolds have similar coverage and GC content (Supplementary Fig. 2). When compared to other parasitoids with a chromosomal scale genome, the assembly statistics are similar in both genome size and N50 length of scaffolds except *Alloplasta piceator* with a larger genome size (549.8 Mbp) and *C. congregata* with a smaller N50 length (1.12 Mb) (Table 2). The chromosome sizes range from 17.33 to 41.1 Mbp with the G + C contents ranging from 38.6 to 40.4% (Table 3). Genome annotation with the NCBI Eukaryotic Annotation Pipeline yielded 14,009 genes or pseudogenes, including 11,831 containing protein-coding regions, and 23,831 annotated mRNA transcripts (Table 4). Gene coding densities vary among chromosomes (Table 3). Evidence for gene annotations were derived from RNA-Seq data in this study (Table 1) and those existing in GenBank as of August 2021, proteins from related species, or ab initio evidence predicted by GNOMON. A large proportion of transcripts [22,626 of 23,831 (94.9%)] were fully supported with experimental evidence. A total of 2,028 noncoding genes, 197 tRNAs, 2,167 lncRNAs and other genome components were also identified (Table 4). Details of the annotation are presented in Tables 3 & 4 as well as online at https://www.ncbi.nlm.nih.gov/genome/annotation_euk/Venturia_canescens/100/.

**Table 2.** Assembly summary statistics compared to other parasitoid genomes.

Species	NCBI BioProject	Contig count (N50 Mb)	Scaffold count (N50 Mb)	Total length (Mb)	GC (%)
Venturia canescens*	PRJNA736740	100 (11.2)	67 (24.99)	290.8	39.6
Alloplasta piceator*	PRJEB55792	1,462 (1.12)	377 (56.99)	549.8	36
Campoletis raptor*	PRJEB58800	177 (2.19)	21 (18.62)	218.6	36.5
Amblyteles armatorius*	PRJEB51578	157 (4.38)	104 (17.13)	227.1	44.3
Ichneumon xanthorius*	PRJEB48052	374 (4.08)	151 (20.14)	315	42.9
Buathra laborator*	PRJEB51796	142 (10.41)	110 (17.03)	329.9	42.6
*Microplitis demolitor*	PRJNA251518	27,508 (0.014)	1,794 (1.14)	241.2	33.1
*Chelonus insularis*	PRJNA624215	457 (1.16)	455 (1.16)	135.7	30.5
Cotesia congregata*	-	- (0.049)	3,140 (1.12)	206.9	28.2
*Cotesia vestalis*	PRJNA271135	9,156 (0.046)	-	186.1	30.6
*Fopius arisanus*	PRJNA258104	8,510 (0.052)	1,042 (0.98)	153.6	39.4
*Diachasma alloeum*	PRJNA306876	25,534 (0.044)	3,968 (0.65)	388.8	39.1
Nasonia vitripennis*	PRJNA13660	25,484 (0.019)	6,169 (0.71)	295.8	40.6

assembled at a chromosomal scale.

**Table 3.** Annotation statistics of each chromosome.

Chrom	Accession	Size (Mb)	GC%	Protein	rRNA	tRNA	Other RNA	Gene	Pseudo
Chrom 1	NC_057421.1	41.1	38.9	3,811	-	35	509	2,138	8
Chrom 2	NC_057422.1	34.83	39.6	2,678	1	11	342	1,536	12
Chrom 3	NC_057423.1	30.39	39.3	1,723	-	13	291	1,113	9
Chrom 4	NC_057424.1	25.1	40.4	2,674	34	33	349	1,583	15
Chrom 5	NC_057425.1	24.99	40.1	2,097	9	26	251	1,247	8
Chrom 6	NC_057426.1	23.18	39.4	1,797	-	5	189	964	8
Chrom 7	NC_057427.1	22.67	39.8	2,312	-	10	241	1,256	6
Chrom 8	NC_057428.1	22.63	39.8	2,013	-	30	196	1,118	12
Chrom 9	NC_057429.1	21.67	39.9	1,892	38	14	178	1,094	5
Chrom 10	NC_057430.1	18.87	40.1	1,523	-	6	136	878	10
Chrom 11	NC_057431.1	17.33	38.6	1,279	-	14	193	828	16

**Table 4.** Gene annotation summary statistics.

Feature	Count	Mean length (bp)	Median length (bp)	Min length (bp)	Max length (bp)
Genes	13,859	14,044	3,980	70	2,962,486
protein-coding	11,831				
non-coding	2,028				
All transcripts	27,171	3,724	2,697	70	59,128
mRNA	23,831	3,971	2,900	243	56,128
misc_RNA	673	3,700	2,560	150	21,243
tRNA	197	74	73	71	84
lncRNA	2,167	1,734	1,226	91	12,367
CDSs	23,831	2,174	1,548	213	57,756
Exons	99,793	465	223	2	19,327
Introns	83,888	2,543	253	30	535,078

An assessment of the completeness of gene annotation using BUSCO shows that 97.3% of the Insecta BUSCOs are present in the *V. canescens* genome assembly. Only 0.3% of those BUSCOs were detected as fragmented. We compared the BUSCO results with those of the braconid genome of *C. congregata* which was also assembled at the chromosomal scale (Gauthier et al. 2021). The comparison shows that the percentage of complete BUSCO genes in the *V. canescens* genome is comparable but marginally higher than that of the *C. congregata* genome, indicating the assembly is highly complete (Table 5).

**Table 5.** BUSCO analysis of the *V. canescens* and *C. congregata* gene annotation completeness.

Species	Gene count	Complete (%)		Fragmented (%)	Missing (%)
		Single-copy	Duplicated		
*V. canescens*	13,859	96.6	0.6	0.3	2.5
*C. congregata*	14,140	88.5	4.5	3.3	3.7

**Fig. 1.**
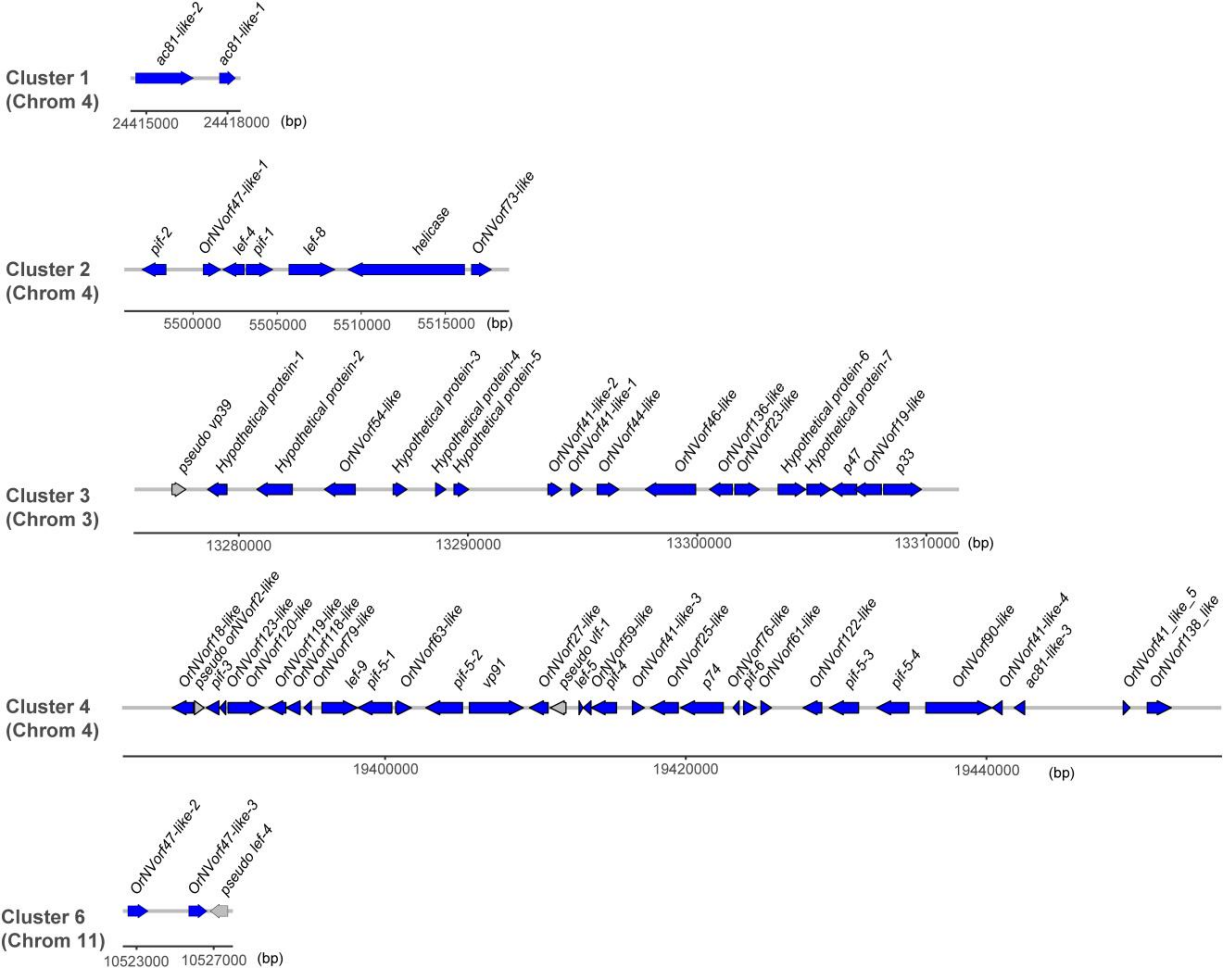
Locations of nudivirus gene clusters in *V. canescens* chromosomes.

Previous characterization of virus-derived components in the *V. canescens* genome revealed that 53 nudivirus genes were organized into six clusters surrounded by wasp genes (Pichon et al. 2015). We identified all 53 genes in the newly assembled genome (Table 6; Fig. 1). In addition, one more copy of the *OrNVorf47-like* gene family (*OrNVorf47-like-6*) was also identified based upon homology with the other five previously annotated copies. Unlike other nudivirus genes, *OrNVorf47-like-6* is not located in any of the nudivirus-derived gene clusters. We further found seven ORFs interspersed within cluster 3 with no similarity to known genes in the NCBI nr database. However, an examination of sequence read coverage from the transcriptome data of ovaries shows that they are transcribed with similar coverage and boundaries compared with the neighboring nudivirus-derived genes in cluster 3. Therefore, we annotated them as hypothetical proteins that are likely of nudivirus origin (Table 6; Fig. 1). None of these newly annotated hypothetical genes had introns except hypothetical protein 6. In addition to intact nudivirus-derived genes, a recent study also identified 19 pseudogenized nudivirus-derived genes, of which homologous genes in baculoviruses have predicted functions that have been lost in VLPs (e.g. capsid components) (Leobold et al. 2018; Drezen et al. 2022). All 19 previously characterized pseudogenes were identified in the current genome assembly.

**Table 6.** Nudivirus genes and pseudogenes identified in the *V. canescens* genome.

Chrom (length, Mbp)	Cluster	Start	End	Gene	Accession	Function	Expression in ovaries (FPKM)
C1_NC_057421.1 (41.1)		13,046,202	13,046,478	*pseudo-OrNVorf139-like*	N/A	Unknown	0
		25,445,192	25,445,817	*pseudo-OrNVorf117-like*	N/A	Nucleocapsid	0
C2_NC_057422.1 (34.83)		22,501,670	22,502,290	*pseudo-38K*	N/A	Nucleocapsid	0
C3_NC_057423.1 (30.39)		7,915,392	7,915,643	*pseudo-OrNVorf128.1-like*	N/A	Nucleocapsid	0
	3	13,277,052	13,277,652	*pseudo-vp39*	N/A	Nucleocapsid	1
		13,278,601	13,279,450	Hypothetical protein-1	KAI5630585.1	Unknown	189
		13,280,751	13,282,300	Hypothetical protein-2	KAI5630586.1	Unknown	93
		13,283,701	13,285,050	*OrNVorf54-like*	KAI5630587.1	Unknown	212
		13,286,701	13,287,300	Hypothetical protein-3	KAI5630588.1	Unknown	623
		13,288,551	13,289,000	Hypothetical protein-4	KAI5630589.1	Unknown	966
		13,289,351	13,289,999	Hypothetical protein-5	KAI5630590.1	Unknown	772
		13,293,451	13,294,050	*OrNVorf41-like-2*	KAI5630591.1	Unknown	474
		13,294,451	13,294,950	*OrNVorf41-like-1*	KAI5630592.1	Unknown	1099
		13,295,601	13,296,550	*OrNVorf44-like*	KAI5630593.1	Unknown	486
		13,297,701	13,299,900	*OrNVorf46-like*	KAI5630594.1	Unknown	39
		13,300,501	13,301,500	*OrNVorf136-like*	KAI5630595.1	Unknown	97
		13,301,601	13,302,670	*OrNVorf23-like*	KAI5630596.1	Unknown	14
		13,303,496	13,304,700	Hypothetical protein-6	KAI5630597.1	Unknown	298
		13,304,751	13,305,800	Hypothetical protein-7	KAI5630598.1	Unknown	935
		13,305,828	13,306,926	*p47*	KAI5630599.1	Transcription	24
		13,306,838	13,308,000	*OrNVorf19-like*	KAI5630600.1	Unknown	27
		13,308,101	13,309,750	*p33*	KAI5630601.1	Nucleocapsid/Envelope	120
		14,394,309	14,394,566	*pseudo-OrNVorf128.2-like*	N/A	Nucleocapsid	0
C4_NC_057424.1 (25.1)		4,383,268	4,383,801	*pseudo-OrNVorf99.2-like*	N/A	Unknown	0
	2	5,496,951	5,498,400	*pif-2*	KAI5630602.1	Envelope	43
		5,500,601	5,501,650	*OrNVorf47-like-1*	KAI5630603.1	Unknown	69
		5,501,751	5,503,050	*lef-4*	KAI5630604.1	Transcription	9
		5,503,151	5,504,750	*pif-1*	KAI5630605.1	Envelope	79
		5,505,701	5,508,450	*lef-8*	KAI5630606.1	Transcription	6
		5,509,201	5,516,150	*helicase*	KAI5630607.1	Replication	3
		5,516,551	5,517,750	*OrNVorf73-like*	KAI5630608.1	Unknown	1
		5,557,092	5,557,427	*pseudo-OrNVorf62-like*	N/A	Unknown	23
		5,593,624	5,595,560	*pseudo-fen-1*	N/A	Replication	0
		5,702,918	5,703,779	*pseudo-OrNVorf9-like*	N/A	Unknown	0
		7,841,691	7,843,015	*pseudo-pif-1*	N/A	Envelope	0
		14,125,555	14,126,938	*pseudo-dnapol*	N/A	Replication	1
		17,556,801	17,557,550	*OrNVorf41-like-6*	KAI5630609.1	Unknown	112
	4	19,385,877	19,387,315	*OrNVorf18-like*	KAI5630610.1	Unknown	222
		19,387,367	19,388,012	*pseudo-OrNVorf2-like*	N/A	Unknown	45
		19,388,151	19,389,000	*pif-3*	KAI5630611.1	Envelope	56
		19,389,050	19,389,450	*OrNVorf123-like*	KAI5630612.1	Unknown	130
		19,389,601	19,392,000	*OrNVorf120-like*	KAI5630613.1	Unknown	73
		19,392,301	19,393,450	*OrNVorf119-like*	KAI5630614.1	Unknown	320
		19,393,455	19,394,400	*OrNVorf118-like*	KAI5630615.1	Unknown	120
		19,394,651	19,395,150	*OrNVorf79-like*	KAI5630616.1	Unknown	206
		19,395,851	19,398,200	*lef-9*	KAI5630617.1	Transcription	42
		19,398,251	19,400,500	*pif-5-1*	KAI5630618.1	Envelope	27
		19,400,751	19,401,800	*OrNVorf63-like*	KAI5630619.1	Unknown	36
		19,402,751	19,405,200	*pif-5-2*	KAI5630620.1	Envelope	26
		19,405,651	19,409,250	*vp91*	KAI5630621.1	Envelope	55
		19,409,650	19,410,900	*OrNVorf27-like*	KAI5630622.1	Unknown	92
		19,411,033	19,412,084	*pseudo-vlf-1*	N/A	Replication/Nucleocapsid	7
		19,412,951	19,413,205	*lef-5*	KAI5630623.1	Transcription	26
		19,413,229	19,413,750	*OrNVorf59-like*	KAI5630624.1	Unknown	363
		19,413,757	19,415,450	*pif-4*	KAI5630625.1	Envelope	200
		19,416,501	19,417,300	*OrNVorf41-like-3*	KAI5630626.1	Unknown	194
		19,417,701	19,419,550	*OrNVorf25-like*	KAI5630627.1	Unknown	90
		19,419,701	19,422,550	*p74*	KAI5630628.1	Envelope	305
		19,423,201	19,423,600	*OrNVorf76-like*	KAI5630629.1	Unknown	66
		19,423,901	19,424,750	*pif-6*	KAI5630630.1	Envelope	65
		19,425,051	19,425,750	*OrNVorf61-like*	KAI5630631.1	Unknown	105
		19,427,851	19,429,100	*OrNVorf122-like*	KAI5630632.1	Nucleocapsid	19
		19,429,600	19,431,550	*pif-5-3*	KAI5630633.1	Envelope	39
		19,432,751	19,434,900	*pif-5-4*	KAI5630634.1	Envelope	105
		19,436,026	19,440,400	*OrNVorf90-like*	KAI5630635.1	Unknown	20
		19,440,451	19,441,100	*OrNVorf41-like-4*	KAI5630636.1	Unknown	67
		19,441,901	19,442,600	*ac81-like-3*	KAI5630637.1	Unknown	88
		19,449,151	19,449,601	*OrNVorf41-like-5*	KAI5630638.1	Unknown	115
		19,450,751	19,452,350	*OrNVorf138-like*	KAI5630639.1	Unknown	82
	1	24,414,601	24,416,750	*ac81-like-2*	KAI5630640.1	Unknown	6
		24,417,701	24,418,300	*ac81-like-1*	KAI5630641.1	Unknown	40
C5_NC_057425.1 (24.99)		14,619,513	14,619,795	*pseudo-OrNVorf130-like*	N/A	Unknown	0
C7_NC_057427.1 (22.67)		8,505,478	8,506,069	*pseudo-OrNVorf22-like*	N/A	Unknown	27
		14,311,928	14,312,841	*pseudo-integrase*	N/A	Replication/Nucleocapsid	0
C9_NC_057429.1 (21.67)		19,449,204	19,449,601	*pseudo-OrNVorf99.1-like*	N/A	Unknown	0
C11_NC_057431.1 (17.33)	6	10,522,551	10,523,600	*OrNVorf47-like-2*	KAI5630645.1	Unknown	7
		10,525,701	10,526,651	*OrNVorf47-like-3*	KAI5630646.1	Unknown	50
		10,526,823	10,527,731	*pseudo-lef-4*	N/A	Transcription	1

**Fig. 2.**
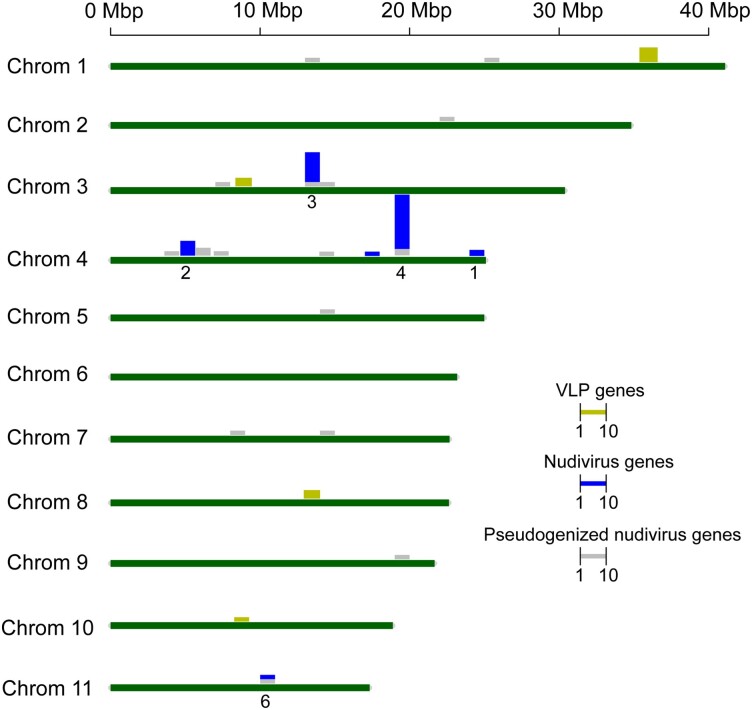
*V. canescens* chromosome map with the distribution of intact and pseudogenized nudivirus-derived genes and VLP genes. Numbers of genes in 1 Mbp-length loci are indicated by vertical bars with coloration and scale indicated in the figure legend. Gene clusters are labeled with numbers.

Having catalogued all previously characterized intact and pseudogenized nudivirus-derived genes in the genome, we next examined where these elements were located in chromosomes. Clusters 1, 2, 4, and 5 were all located in chromosome 4 (Figs. 1 & 2), while clusters 3 and 6 were located in chromosome 3 and 11, respectively. We further detected that clusters 4 and 5 are adjacent and form a single cluster that we named cluster 4. We did not re-name cluster 6 to be consistent with how it had been named earlier (Pichon et al. 2015). Four pseudogenes (*pseudo-vp39*, *pseudo-OrNVorf2-like*, *pseudo-vlf-1*, and *pseudo-lef-4*) were located in clusters 3, 4, and 6, and the remainder were dispersed among 8 chromosomes (Fig. 2).

Genes for *VLP1-3* are located far from the nudivirus-derived gene containing clusters. *VLP1* and *VLP3* are in chromosomes with no nudivirus-derived gene clusters. While *VLP2* is located in chromosome 3, which also contains nudivirus-derived gene cluster 3, *VLP2* is located about 4Mb away from that cluster. *VLP2* and *3* each have paralogs located next to them (Table 7; Fig. 2). In *V*. *canescens* ovaries, *VLP2* is expressed about twice as much as its paralog, and *VLP3* is expressed about 160 times as much as its neighboring paralog. Additionally, other genes similar to *VLP3* were identified in chromosome 1, but these were expressed at low levels in the ovaries.

**Table 7.** VLP genes identified in the *V. canescens* genome.

Chrom (length, Mbp)	Start	End	Gene	Accession	Predicted function	Expression in ovaries (FPKM)
C1_NC_057421.1 (41.1)	36,504,557	36,524,198	*VLP3p-2*	KAI5630565.1	neprilysin-like	30
	36,526,558	36,533,880	*VLP3p-3*	KAI5630568.1	neprilysin-like	10
	36,534,085	36,543,403	*VLP3p-4*	KAI5630573.1	neprilysin-like	23
	36,552,978	36,556,446	*VLP3p-5*	KAI5630579.1	neprilysin-like	0
	36,560,156	36,566,977	*VLP3p-6*	KAI5630581.1	neprilysin-like	0
C3_NC_057423.1 (30.39)	9,332,883	9,338,326	*VLP2p-1*	KAI5630583.1	rac GTPase-activating protein 1-like	414
	9,339,477	9,345,184	*VLP2*	KAI5630584.1	rac GTPase-activating protein 1-like	876
C8_NC_057428.1 (22.63)	13,692,502	13,697,475	*VLP3p-1*	KAI5630642.1	neprilysin-like	11
	13,700,752	13,705,772	*VLP3*	KAI5630643.1	neprilysin-like	1763
C10_NC_057430.1 (18.87)	8,910,988	8,913,043	*VLP1*	KAI5630644.1	glutathione peroxidase 1-like	726

Knowledge about the gene content of nudivirus-derived genes in wasp genomes can help ascertain how they arrived there. Dispersed viral genes could be the product of integration of the ancestral viral genome in a single location followed by dispersal, or alternatively integration of multiple copies of the ancestral genome into several locations (Strand and Burke 2013). In both cases, genes may remain intact or become pseudogenized due to a lack of functional constraint if they are not necessary for virion or VLP production. The likely number of ancestral genome copies in the wasp genome can be determined by counting whether most virus-derived genes are present as single or multiple copies; however, this is only possible for cases in which degradation of inactivated (pseudogenized) genes has not proceeded to a point of being unrecognizable. In *V. canescens*, the presence of numerous pseudogenes makes estimation of ancestral genome copies possible. Most nudivirus-derived genes and pseudogenes in the *V. canescens* genome are present as single copy genes except *pif-5*, *ac81-like*, *OrNVorf41-like*, *OrNVorf47-like*, *lef-4*, and *pif-1* gene families. Many of these gene family members most likely represent localized duplications, as paralogs are often located in clusters, *e.g. pif-5* and *OrNVorf41-like* paralogs in cluster 4. Other paralogs are present in multiple locations, for example, the *OrNVorf47-like* and pseudo-*lef-4* genes in cluster 6 are repeated in the same order and orientation in cluster 2, in which *OrNVorf47-like* was duplicated again and maintained while *lef-4* became pseudogenized. Despite these exceptions, it appears that VcVLPs arose from a single integration event from an ancestral genome because the majority of virus-derived genes are single copy in *V. canescens*.

Examination of the introduction of a set of foreign genes simultaneously into a eukaryotic genome provides the opportunity to study patterns of gene movement and maintenance over time. The *V. canescens* genome assembly at a chromosomal scale provides a comprehensive architectural view of the virus-derived components that produce VcVLPs in the wasp genome, allowing us to investigate the origin and fate of nudivirus-derived genes among wasp chromosomes. If the nudivirus-derived genes in the *V. canescens* genome arose from a single integration event, it follows that the ancestral circular double-stranded DNA viral genome must have become linearized and integrated into a single locus in the wasp genome originally. Our chromosome-scale assembly confirms the previous finding that *V. canescens* nudivirus genes are not widely distributed throughout the wasp genome (Pichon et al. 2015). Currently, all of the nudivirus-derived genes are distributed among 3 chromosomes (out of 11 total), with 3 gene clusters comprising 68% of nudivirus-derived genes located on a single chromosome (chromosome 4). The extent of dispersal of nudivirus-derived genes in wasp genomes can be an indicator of the relative age of viral integration events. Nudivirus-derived gene dispersal in *V. canescens* can be put into context by comparison with other, independently derived DEVs in parasitoid wasps. *Fopius arisanus* is an opiine braconid wasp that has an alphanudivirus-derived DEV that produces VLPs (Burke et al. 2018). It is not known when the alphanudivirus ancestor integrated into the ancestor of *F. arisanus*, but given that nudivirus-derived genes could only be detected in 4/6 species in the genus *Fopius*, it seems likely that this virus is a relatively recent acquisition (Burke et al. 2018). In *F. arisanus,* the nudivirus-derived genes are thought to have arisen from a single integration event and are located in nine clusters throughout the *F. arisanus* genome (Burke et al. 2018).

Fossil evidence suggests BVs have an ∼100my history with parasitoid wasps in the “microgastroid complex” of braconid wasps, representing ∼50,000 extant species (Whitfield 2002; Bézier et al. 2009; Thézé et al. 2011). The nudivirus-derived genes that produce CcBV identified in the *C. congregata* genome have spread among all ten wasp chromosomes and only a single virus gene cluster remains (Gauthier et al. 2021). In another BV-producing species, *C. insularis,* no large clusters (>5 genes) of virus-derived genes remain (Mao et al. 2022). Overall, the organization of nudivirus genes in the *V. canescens* chromosomes suggests that the acquisition of the viral ancestor in this species happened within a more recent or similar time frame to the DEV of *F. arisanus,* and much more recently when compared to the age of BVs in microgastroid wasps (Drezen et al. 2017; Burke et al. 2021).

A chromosomal assembly of the *V. canescens* genome now also makes it possible to examine the maintenance or loss of nudivirus-derived genes over time in relation to their locations in the wasp genome. Unlike BV-producing wasps with few recognizable pseudogenized nudivirus-derived genes, several nudivirus-derived genes encoding nucleocapsid components and genes with unknown function were pseudogenized in the *V. canescens* genome (Leobold et al. 2018). Due to their presence in clusters with other, intact, nudivirus-derived genes, it is likely that three genes (*pseudo-vp39*, *pseudo-OrNVorf2-like*, and *pseudo-vlf-1*) were pseudogenized in place. In contrast, *pseudo-lef-4* and *pseudo-pif-1* seem to have duplicated and dispersed in chromosomes 4 and 11, followed by pseudogenization (with an intact copy of both genes remaining in cluster 2). The remaining 14 pseudogenes are dispersed among eight chromosomes and are not associated with nudivirus gene clusters. As most do not have paralogs in the *V. canescens* genome (intact or pseudogenized), this is suggestive of a link between the processes of dispersal and pseudogenization. While the pseudogenization of genes in place is most likely related to a lack of functional constraint for their role in making components lacking in VLPs (such as nucleocapsid components), pseudogenization via dispersal could sometimes be a random process unrelated to functional constraint early after the acquisition of an ancestral viral genome. The random dispersal and pseudogenization of a gene encoding one of the essential nucleocapsid components (*e.g. 38K*) could be the event that resulted in *V. canescens* producing VLPs rather than nucleic-acid containing virions. The early dispersal of nudivirus-derived genes in wasp genomes could have important functional consequences, making the reconstruction of rearrangement events between related species that produce VLPs or viruses of interest for future studies.

Finally, it has been suggested previously that the VLPs produced by *V. canescens* replaced an ichnovirus association because *V. canescens* was assumed to belong to a clade of wasps that all produce ichnoviruses (Pichon et al. 2015). However, evidence for the remnants of ichnovirus genes in the *V. canescens* genome is weak (Pichon et al. 2015) and this species may not belong to an ichnovirus-producing clade of wasps (Burke et al. 2021). The high-quality genome assembly for *V. canescens* will allow for future comparative genomic studies of closely related species to elucidate the nudivirus acquisition and domestication process and any potential ichnovirus replacement in this wasp species and relatives.

## Supplementary Material

jkad137_Supplementary_Data

## Data Availability

All raw HiFi, HiC, and RNA-Seq read datasets are available from the NCBI SRA database (see Materials and Methods, and Table 1 for accessions). The genome assembly, WGS Project JAHMHP01, is represented as BioProject PRJNA736740 and BioSample SAMN19659032 with identical records in GenBank as accession GCA_019457755.1 and RefSeq as accession GCF_019457755.1 named ASM1945775v1. An FTP site for data download is at ftp.ncbi.nlm.nih.gov/genomes/all/annotation_releases/32260/100/. NCBI's Genome Data Viewer can be accessed at https://www.ncbi.nlm.nih.gov/genome/gdv/browser/genome/?id=GCF_019457755.1 and an overview of release 100 annotations can be accessed at https://www.ncbi.nlm.nih.gov/genome/annotation_euk/Venturia_canescens/100/. Curation of this assembly and consolidated sequence-based resources are hosted by the i5k Workspace (https://i5k.nal.usda.gov/) allowing visualization within jBrowse, manual curation with Apollo and other tools. Supplemental material available at G3 online.
