# Supplementary figures and images for: A chromosome scale assembly of the parasitoid wasp Venturia canescens provides insight into the process of virus domestication
Source: G3 (Bethesda). 2023 Jun 22;13(10):jkad137. doi: 10.1093/g3journal/jkad137 (PMC10542567; doi:10.1093/g3journal/jkad137)

Figure S1 BlobPlot of the *V. canescens* genome assembly.

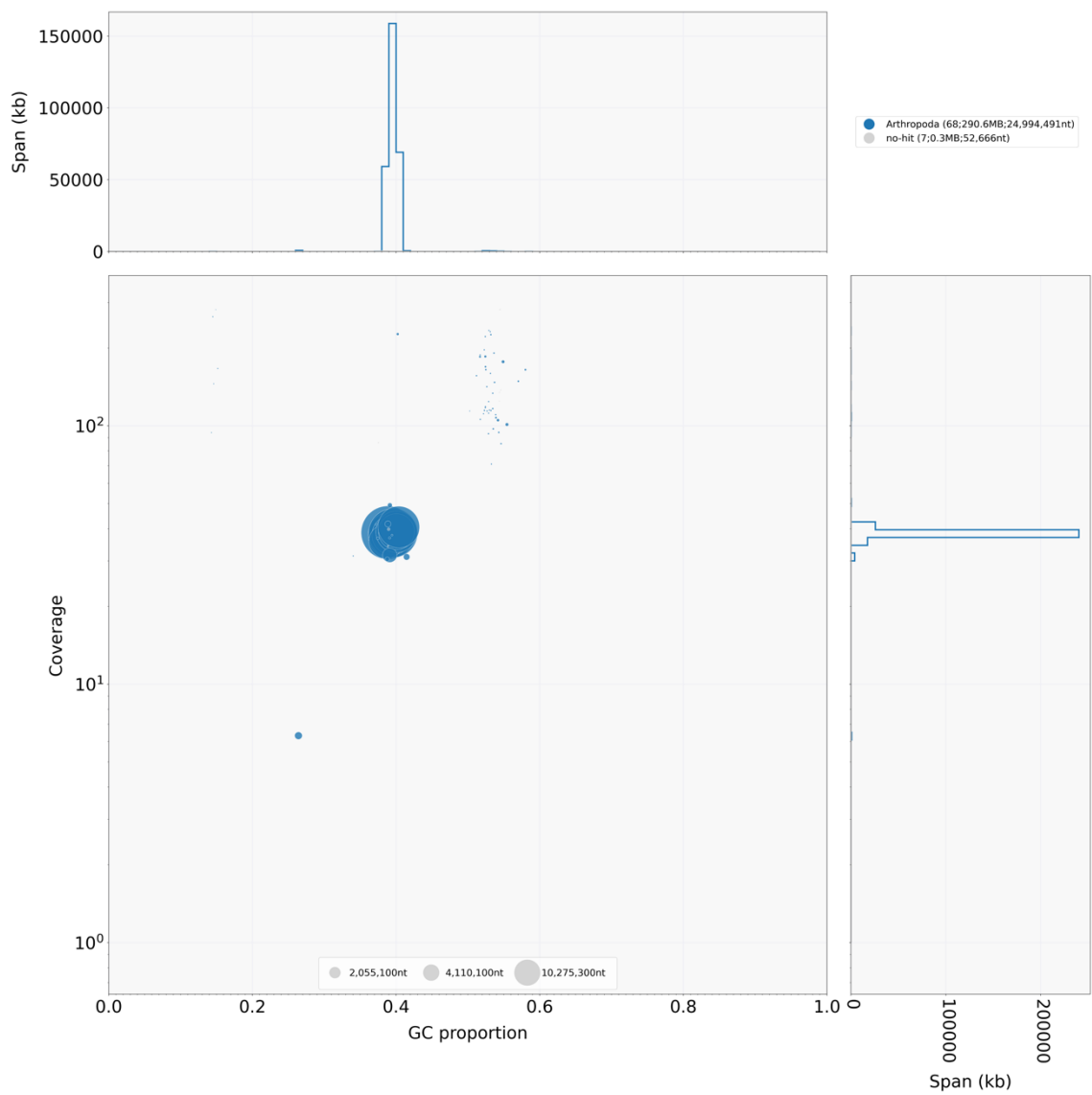

Supplement: jkad137_Supplementary_Data [file jkad137_supplementary_data.zip › Figure_S1_G3-2023-404054.pdf]

Figure S2 HiC contact map of the *V. canescens* genome assembly.

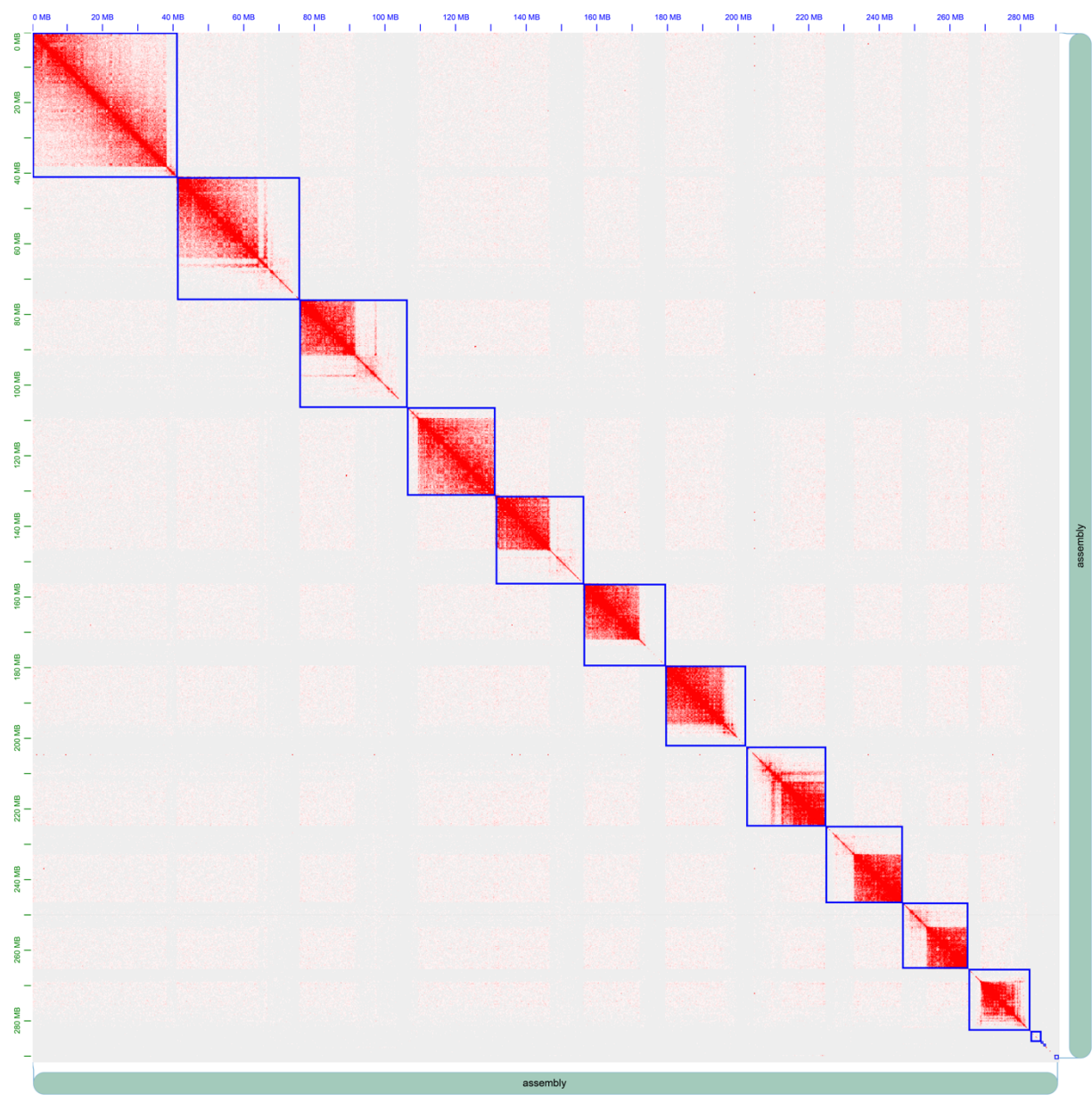

Supplement: jkad137_Supplementary_Data [file jkad137_supplementary_data.zip › Figure_S2_G3-2023-404054.pdf]
